# Visual homeostatic processing in V1: when probability meets dynamics

**DOI:** 10.3389/fnsys.2015.00006

**Published:** 2015-02-03

**Authors:** Nora Nortmann, Sascha Rekauzke, Zohre Azimi, Selim Onat, Peter König, Dirk Jancke

**Affiliations:** ^1^Optical Imaging Group, Institut für Neuroinformatik, Ruhr University BochumBochum, Germany; ^2^Institute of Cognitive Science, University of OsnabrückOsnabrück, Germany

**Keywords:** visual cortex, predictive coding, homeostasis, information transmission, orientation difference detection, natural image processing

Adaptation and homeostasis, the ability to reach stable attractor states within changing environments, are the most typical characteristics of biological systems. A recent study by Benucci et al. (2013) reported that the primary visual cortex (V1) counteracts biases in a rapidly changing stimulus ensemble by introducing the appropriate opposing biases in the responsiveness and selectivity of neurons. Using sequences of differently oriented gratings (32 ms frame duration) and biasing the input statistics toward one orientation (the “adaptor”), such that the selected orientation occurred three to four times more often than all other orientations, a remarkable adaptive behavior was found: decreased activities in response to the adapter were exactly counterbalanced such that the average population signal was kept constant. This was attributed to homeostatic mechanisms “that work toward two simple goals: to maintain equality in the time-averaged responses across the population and to enforce independence in selectivity across the population.” When calculating the necessary time span to show these effects, the authors concluded that V1 needs ~1.7 s in order to catch up with the actual statistical input properties and, thus, adapt adequately to the bias within the sequence of input.

While it is an intriguing idea, assigning V1 a role as a probability detector that integrates incoming information over a considerable time of multiple seconds, our own data (Nortmann et al., 2013) revealed a much smaller time window for a similar effect; that is, responses to the adapting orientation and to all other orientations balanced each other within 100 ms (Figure 1A, hatched areas in bottom graph). In this study, we used voltage-sensitive dye imaging to capture V1 population dynamics and applied unbiased 10-Hz sequences of oriented stimuli. Moreover, our data suggest that this mechanism is effective for gratings as well as for natural stimuli, and even for single isolated switches. In the example shown, vertically and horizontally filtered natural images were presented, depicted here as gratings for simplicity. Because superimposed orientations were also embedded (cf. plaid in Figure 1A marked purple) in our sequences, a switch from a single orientation to superposition could be analyzed with respect to its underlying adapting component (marked red) and the newly added orientation (i.e., the changing component, blue). Comparing the calculated superimposed component responses with the measured superposition responses revealed that population tuning amplitude for adapted orientation decreased (see purple line and downward arrow) while activity of the changing orientation was facilitated in the opposite direction (upward arrow). Strikingly, the effect was also valid for switches in opposite direction; that is, when a single orientation was removed from superposition, most likely due to increased contributions from OFF responses. In addition, these results suggest that V1 encoded orientation differences rather than current orientations (see Eriksson et al., 2012 for similar findings) and hence, reduced input redundancies in accordance with predictive encoding principles (Rao and Ballard, 1999). These immediate dynamics might be mediated by tuned “push–pull”-like mechanisms (Hirsch et al., 2003) involving synaptic depression (Nelson, 1991) and post-inhibitory rebound (Creutzfeld and Struck, 1962; Sanchez-Vives et al., 2000).

**Figure 1:**
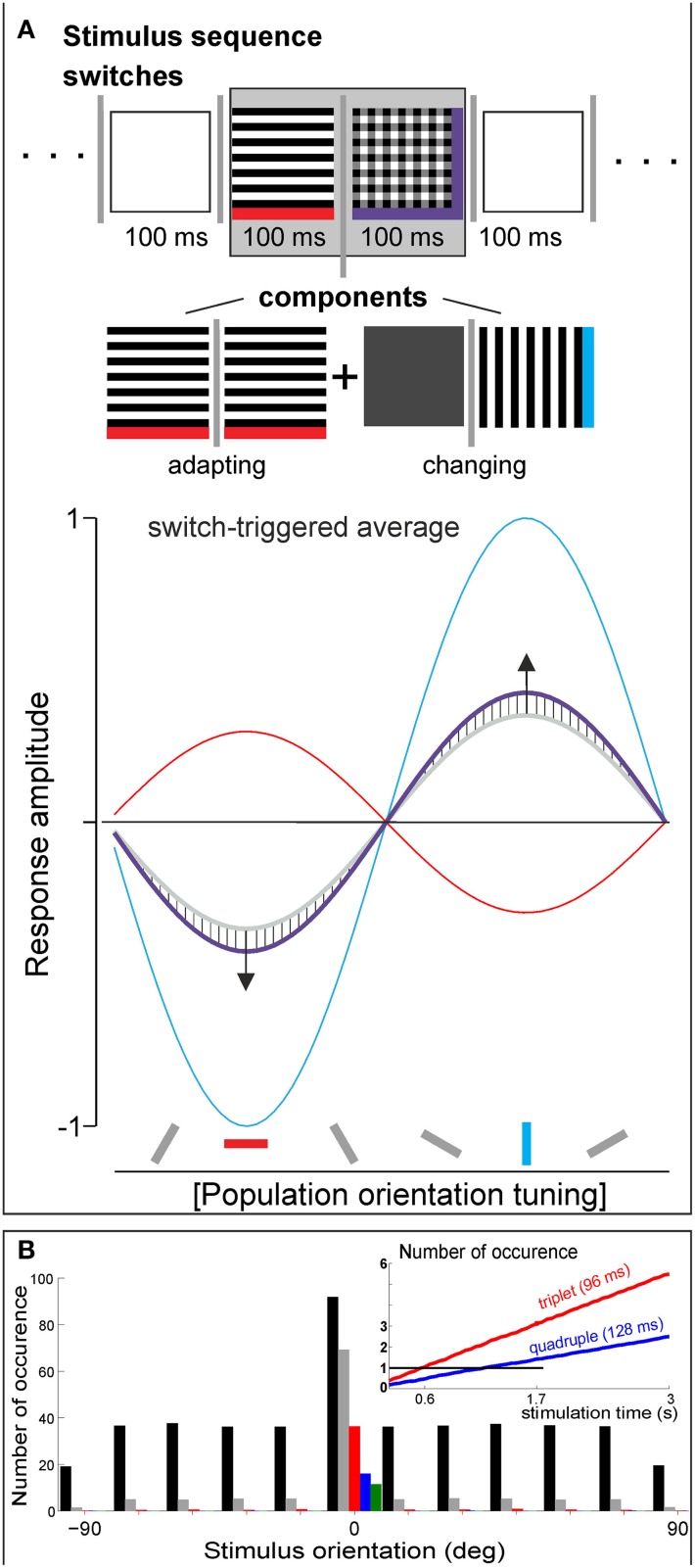
**(A)** In Nortmann et al. (2013), pseudorandom stimulus sequences of 17 stimuli (vertically and horizontally filtered natural images, their superpositions, and isoluminant gray image) were presented at 10 Hz (>64 shuffled repetitions). For switch-triggered averaging, sequences were aligned to a specific switch between a pair of stimuli, here to a switch from a single orientation to superimposed horizontal and vertical orientations (see sketch in gray box at top). Plot depicts fitted V1 population tuning curves for adaptive component (red), changing component (blue), and composite switch (purple curve, median across 12 different experiments). Hatched areas indicate deviations (~20%) from the component average (gray). **(B)** Introducing a bias in one orientation (“adapter”) across random sequences of differently oriented gratings. A simple permutation test was done: adapter bias was set to 30% probability, 12 different orientations were simulated (100 repetitions), single frame = 32 ms, overall stimulation time was 20 s (#625 frames), as used in Benucci et al. (2013). Number of adapter occurrences as single, doublet, triplet, quadruple, and quintuple (black, gray, red, blue, and green, respectively). Inset: number of counts (black horizontal line represents *N* = 1) for 96 and 128 ms periods of adapter stimulation after 3 s of sequence presentation. After 0.6 s, probability is enhanced to include at least one triplet (red; cf. start of adaptation effect in Supplementary Figure 5 of Benucci et al., 2013). After 1.7 s, a presence of quadruple is likely (blue); variance was smaller than line width.

Although the stimulation protocols in the studies outlined here have differed in several aspects, we think a link between them exists. The biased stimulation protocol in Benucci et al. (2013) inevitably promotes occurrence of doublets, triplets, etc. and thus repeated frames, leading to longer adapter durations of effectively 64, 96 ms etc., rather than independent presentations of single adapter frames (Figure 1B). Particularly, the onset of adaptation may vary with the introduced amount of statistical bias. Hence, the reported 1.7 s window might comprise a stimulation-specific time span needed for collecting enough responses to long-duration adapters to reach the signal detection threshold (Figure 1B, inset) within the particular regime of boundary conditions. To be conclusive however, several open questions remain to be addressed in future experiments. For example, in Benucci et al. (2013), adaptation occurs exponentially, whereas probability for long duration adapters increases linearly. Thus, it is not clear if the counterbalancing effects, as observed in Nortmann et al. (2013), are of sufficient magnitude to explain homeostasis as found by Benucci et al. (2013). Moreover, in Benucci et al. (2013) the phase of the gratings was varied while Nortmann et al. (2013) used stationary images. The latter could promote contributions of simple cells, whereas variation in phase averages these out and may enhance nonlinear complex cell contributions.

Regardless of the underlying neural mechanisms: which benefits are provided by cortical adaptation in the hundreds of milliseconds up to second ranges? One may ask the question the other way around: which disadvantages are brought about by longer adaptation times? As often, the answer depends on the task. We suggest that this is when eye movements come into play. For inspection of fine details within natural scenes, high-frequency sampling is useful (Rucci et al., 2007), requiring ongoing cortical encoding (Benucci et al., 2009; Nortmann et al., 2013), and rapid transmission of information along with further adaptive cortical mechanisms acting at tens of milliseconds (Felsen et al., 2002). In contrast, for saccades occurring at much lower frequencies and on larger spatial scales, it may be advantageous to emphasize stimulus differences to past input (Movshon and Lennie, 1979; Müller et al., 1999; Dragoi et al., 2000), as natural scene statistics predict distant image structure sampled by saccades, to be weakly correlated (Dragoi et al., 2002).

In the study by Benucci et al. (2013), the dense sampling over different orientations (up to 12) allowed a comprehensive modeling account for cortical homeostasis and decorrelation effects across fine-scaled orientation space. In our work (Nortmann et al., 2013), we showed counterbalancing effects already after 100 ms adapter times, without probabilistic accumulation of external stimulus statistics over seconds. In fact, the underlying internal processing dynamics may have been implemented in neuronal functioning via adaptation to embodied sensorimotor regularities, such as provided by eye movements. As emphasized in Benucci et al. (2013), adaptation operates on multiple timescales (Wark et al., 2007; Haak et al., 2014). In this way, indeed, input statistics experienced during a lifetime may guide manifold cortical network properties while “homeostasis” acts as a dynamic attractor that maintains the ability of the cortex to perform lively deviations from baseline across populations of neurons.

## Conflict of interest statement

The authors declare that the research was conducted in the absence of any commercial or financial relationships that could be construed as a potential conflict of interest.
